# Measuring Risk Perception in Pregnant Women in Heavily Polluted Areas: A New Methodological Approach from the NEHO Birth Cohort

**DOI:** 10.3390/ijerph182010616

**Published:** 2021-10-11

**Authors:** Silvia Ruggieri, Sabina Maltese, Gaspare Drago, Simona Panunzi, Fabio Cibella, Fabrizio Bianchi, Fabrizio Minichilli, Liliana Cori

**Affiliations:** 1 National Research Council of Italy, Institute for Biomedical Research and Innovation, 90146 Palermo, Italy; silvia.ruggieri@irib.cnr.it (S.R.); sabinamaltese@libero.it (S.M.); fabio.cibella@irib.cnr.it (F.C.); fabriepi@ifc.cnr.it (F.B.); 2 National Research Council of Italy, Institute for System Analysis and Computer Science—BioMatLab, 00168 Rome, Italy; simona.panunzi@biomatematica.it; 3 National Research Council of Italy, Institute of Clinical Physiology, 56124 Pisa, Italy; fabrizio.minichilli@ifc.cnr.it (F.M.); liliana.cori@ifc.cnr.it (L.C.)

**Keywords:** risk perception, industrially contaminated sites, birth cohort, pregnancy, risk communication

## Abstract

Risk perception (RP) evaluation during pregnancy and its relationship with lifestyles are considered useful tools for understanding communities living in high-risk areas and preventing dangerous exposure. It is well known that exposure to pollutants and less-healthy lifestyles may result in increased disease occurrence during life. Our work investigated environmental RP through ad hoc questionnaires administered to 611 mothers within the NEHO birth cohort, recruited in three heavily contaminated areas of Southern Italy. Four different RP indices, an exploratory factorial analysis (EFA), and a latent class analysis were evaluated from questionnaires. The highest values of risk perception index were observed in the Milazzo site (0.64 ± 0.16) and the lowest in the Crotone site (0.5 ± 0.18). EFA revealed four latent factors, including different items describing environmental pollution, and subjects were classified into four latent classes with different RP indices. Significant RP profiles were different among the sites (*p* < 0.001). Our results did not demonstrate any association between RP and lifestyles during pregnancy. Improving healthy lifestyle behaviours, particularly in polluted areas, would generate co-benefits by preventing further risk factors. As remediation interventions can take a long time, it needs to improve healthy lifestyles in residents until remediation is completed.

## 1. Introduction

The relationship between industrial pollution and human health is of extreme relevance for public health. Living in proximity to industrially contaminated sites (ICSs) and being exposed to increasing concentrations of environmental pollutants, along with disadvantaged social and economic conditions, result in an increased occurrence of diseases during both childhood and adulthood [1,2,3]. There are many ICSs in Europe, and 42 ICSs in Italy have been defined by law as national priority contaminated sites (NPCSs) for environmental remediation since 1998.

The European Environment Agency [4] has also certified that air pollution, noise, bad smells, and traffic have a severe impact on a population’s health, and that human activities (mainly in the sectors of industry, energy, and transport) produce relevant environmental pressures [5,6,7].

Pregnant women living in highly contaminated sites can be exposed through distinct pathways, and children’s health may be affected by a wide range of pollutants [8,9]. Persuasive epidemiological evidence supports the adverse impact on children’s health caused by exposure to pollutants during early life, as measured during pregnancy and/or childhood [10,11].

Over the last 20 years, the use of human biomonitoring to assess the impact of environmental exposure has been increasing in Europe, particularly in industrialised areas with recognised heavy metal pollution of soil and water [12,13,14,15]. Among diverse factors to consider when studying the impact of pollution on human health, the risk perception (RP) of citizens and communities should be incorporated in the practice of human biomonitoring research in order to decrease environmental exposure and improve risk communication [16]. Thus, RP is an essential element of effective risk communication and for improving risk management programmes [17].

In human biomonitoring research, studies on mothers’ RP usually refer to risks directly related to motherhood and childbirth, exploring particular aspects such as attitudes to vaccination, postpartum depression and anxiety, and perception of pain. In most of these works, the concept of perception is used as a synonym for awareness, recognition, discernment, and understanding [18,19,20,21,22].

This work investigates the RP of the mothers in the Neonatal Environment and Health Outcomes (NEHO) birth cohort [23,24], related to environmental and health issues, examining the influences of various factors such as measured exposure to hazards, educational level, and perception of personal conditions during pregnancy. Mother–child cohort studies are a suitable tool, and likely the most advanced type we can use at present for identifying both maternal and child exposure in early life and possible long-term health outcomes in highly contaminated sites [25].

The NEHO study was activated in the framework of the CISAS (International Centre of Advanced Study in Environment, Ecosystem and Human Health) project, which investigated environmental pollution and its impact on ecosystems and human health [26,27,28,29,30] in three selected NPCSs: Milazzo–Valle del Mela (hereafter referred to as Milazzo) and Augusta–Priolo (hereafter referred to as Priolo), located in the Region of Sicily, and Crotone, located in Calabria. 

Priolo is one of the largest active refinery production sites in Europe, and several disused petrochemical plants with significant residual pollution are also present at the site. Similar oil refinery and power plants are present in the Milazzo site, along with abandoned plants that include asbestos-processing plants [31]. The Crotone area is characterised by three disused industrial areas, in particular pesticide plants, which operated until the 1990s [32].

Several studies have shown that the presence of environmental factors with high visual impact (e.g., refineries, petrochemical and cement plants, power plants) is associated with community feelings of outrage and perceived risk [33]. Previous studies have also shown that individuals with higher perceived risks are more likely to adopt individual health protective measures [34].

To our knowledge, no results of specific studies have been published to date on the RP associated with the living environment and health in mother–child cohorts residing in sites with well-known environmental contamination.

The aim of this study was (i) to evaluate environmental and health RP in pregnant women of the NEHO birth cohort, using RP indices [35,36], defining exploratory factorial analysis (EFA) indices, and adopting a latent class analysis (LCA), and (ii) to determine whether pregnant women with higher perceived risks adopt healthier behaviours than those with lower RP.

## 2. Materials and Methods

Between January 2018 and January 2020, the NEHO cohort enrolled, on a voluntary basis, 845 pregnant women living in the 3 NPCSs of Crotone, Priolo, and Milazzo in the Mediterranean area of Southern Italy, along with pregnant women living in surrounding areas (local reference areas, LRAs), outside the perimeter of the NPCSs but presenting similar geographic and socio-demographic characteristics [25]. Women living in LRAs represent the control group. The study has been conducted following the Declaration of Helsinki. All the adopted procedures comply with the General Data Protection Regulation (UE 2016/679) and Italian laws concerning data protection.

### 2.1. Risk Perception Questionnaire

After enrolment in the NEHO cohort study, mothers were asked to fill out a questionnaire collecting information on maternal health and lifestyle during the gestational and pre-gestational periods. Information on pregnant women’s RP was also collected by means of a questionnaire used in several human biomonitoring surveys [7,35,37,38].

In the present work, we analysed a subset of questions from the J section of this questionnaire, composed of 14 questions about RP, requiring answers on a Likert scale (from 0 to 4) or an answer expressing the “presence” or “absence” of a certain risk. Some of these questions were used to compute RP indices (the selected questions are reported in Supplementary Table S1 and indicated with the letter J). These questions examined the perception of being exposed to air, water, and food pollution, as well as to smells, noise, and to the presence of dangerous industries. The perception of the presence of diseases related to environmental pollution, such as infertility, chronic respiratory disease, allergies, and various types of cancer, was also explored. In the process of evaluating the exposure RP, three additional questions were analysed to evaluate the presence of vehicular traffic and its relative impact on air quality (indicated with the letter E in Table S1).

### 2.2. Risk Perception Indices

The questions included in the J section of the questionnaire were used to compute the four RP indices according to the indications and the formulas reported in previous works [35,36,39]. A total number of 22 items were involved in the indices’ construction. The original formulation was modified to obtain a value for each enrolled mother.

The computed RP indices were:

- the hazard perception index (HPI);
- the exposure hazard perception index (EHPI);
- the health risk perception index (HRPI);
- the risk perception index (RPI).

HPI indicated the mothers’ perception regarding the presence or absence of certain hazards in their residence area. EHPI reflected the questions about the perceptions that mothers had of being exposed to a certain danger in the area where they live. HRPI investigated the perception of the health risk, namely whether, in the participant’s opinion, certain diseases such as allergies, chronic respiratory diseases, infertility, and various types of cancer could arise in the area where they live. Finally, RPI includes the overall perception of both environmental and health risks, taking the different perceptions described above together (Table S1).

For each participant, the HPI, EHPI, HRPI, and RPI were calculated with a value from 0 to 1. The Spearman rank correlation test was used to check for possible pairwise correlations between each pair of indices.

### 2.3. Exploratory Factorial Analysis Indices

Aimed at verifying whether additional information beyond the risk perception definition would be better able to discriminate between participants from contaminated sites and LRAs, an EFA involving a larger number of items, for a total of 28, was used to automatically define a set of four “latent factors” (indices) dependent on the included items. A global index of the perception of risk, EFAtot, was defined as the sum of the four factors.

To evaluate the “factorability” of the data, the Kaiser–Meyer–Olkin test (KMO) and Bartlett’s test were computed. The KMO test measures the sampling adequacy (sample of included variables) in terms of variable correlations. Higher values indicate that a factor analysis can be performed; conversely, a value of less than 0.60 indicates that the sample is not adequate. Bartlett’s sphericity test on the correlation matrix was used to determine whether the included items were unrelated, giving insight into the appropriateness of the procedure. A P value of less than 0.05 indicated that a factor analysis could be carried out with the present data. 

A maximum likelihood approach was used to estimate the factorial loadings, and an oblique rotation method (PROMAX) was used to take correlation among factors into account. The goodness of fit of the factor analysis was evaluated by means of root mean squared error of approximation (RMSEA) and the Tucker–Lewis index (TLI). An RMSEA value lower than 0.05 indicates a minimal approximation error. A very good TLI is obtained for values greater than 0.95. Each factor was built by considering only the items for which the item loadings associated with the factor were the largest in absolute terms. For each mother, the factor value was computed by weighting her responses to the items constituting the factor with the item loadings. All the values were normalised to vary between 0 and 1.

Continuous variables are reported as mean ± SD, while categorical variables are presented as numbers and percentages. For continuous variables and the computed indices, one-way ANOVA was used to test for possible differences among women living in the three NPCSs of Priolo, Milazzo, and Crotone. A t-test and a Wilcoxon test were used to test differences between at-risk areas and LRAs. A chi-squared test or Fisher’s exact test, when appropriate, was used to study the associations between “site” (NPCS) and categorical variables.

Beta regression models were employed for evaluating the relationships between indices and quantitative or qualitative predictors. The class of beta regression models is a generalisation of the logit models in case the response is continuous in the interval (0,1), based on the assumption that the response is beta distributed [40]. In order to evaluate whether higher perceived risks influence healthier behaviour, lifestyle variables (such as smoking, alcohol consumption, physical activity, weight gain, and BMI) were evaluated in a logistic or linear model with each of the computed indices as dependent variables.

### 2.4. Latent Class Analysis

An LCA was used to discover underlying response patterns and groups of respondents with similar characteristics identifying the questionnaire items that (i) best described RP in highly industrialised areas, and (ii) in this context, allowed the characterisation from a geographical and socio-demographic perspective. We selected an optimal subset of items, excluding redundant and non-informative variables for subject classification. Furthermore, following the hypothesis that RP could play a role as a health determinant or health effect modifier, we evaluated whether pregnant women with higher perceived risks adopted healthier behaviours than those with lower RP.

All the analyses were performed in R [41].

A full description of the methods used is provided in the Supplementary Material.

## 3. Results

### 3.1. Socio-Demographic Information

Out of the 845 women who enrolled in the NEHO cohort, 713 participants completed the baseline questionnaires, collecting information on maternal health and lifestyle during the pre-gestational period. Of these, 611 women (406 (66.5%) from the Priolo site, 121 (19.8%) from Crotone, and 84 (13.7%) from Milazzo) answered the questions in the RP J section and were included in the present study.

Table 1 and Table 2 report relevant quantitative and qualitative characteristics, respectively, of the sample of mothers included in the analyses. Mean age was 31.5 ± 4.9 yrs, and the difference among the three sites was borderline (*p* = 0.049), with slightly older mothers in the Milazzo site. Marital status (married, never married, divorced/separated) was not significantly different (*p* = 0.44), mimicking the distribution of the total sample, with the largest percentage of women married (65.5%), followed by never married (33.5%) and divorced/separated (1%). The distribution of educational level (Table 2) was different in the three sites (*p* = 0.02): we found the largest percentage of graduated mothers in Milazzo (41.6%) compared to Crotone (32.8%) and Priolo (27.2%). Moreover, in the Milazzo area, the NPCS and LRA were significantly different, with a larger percentage of women with higher educational levels in the LRA (28% vs. 61.7%, *p* < 0.01).

The crowding index reflects the educational level, even if the index was not significantly different among the three sites (*p* = 0.37): the highest value was recorded in Priolo (1.16 ± 0.56).

### 3.2. Risk Perception Indices

Average values of the RP indices HPI, EHPI, HRPI, and RPI are reported in Table 3. They were all significantly different among the three sites, with the highest values for all the indices observed in Milazzo. The lowest values were observed in Crotone (see Table 3): while within the sites of Priolo and Milazzo, there was a significant difference between at-risk areas and LRAs, in Crotone, only the HPI was significantly differentiated between the two types of areas (0.38 ± 0.23 and 0.25 ± 0.18 in the at-risk areas and in the LRAs, respectively, *p* < 0.01).

### 3.3. Exploratory Factorial Analysis Indices

The EFA highlighted the presence of four “latent factors” (indices). The KMO test (0.91) and Bartlett’s test (*p* < 0.05) indicated the appropriateness of the EFA. The RMSEA and TLI were 0.08 and 0.9, respectively, showing good adaptation of the model to the data.

- Factor 1 (FCT1) corresponds, in terms of building items, to the HRPI index, evaluating the health aspects of the perceived risk.
- Factor 2 (FCT2) includes the items measuring the degree of risk perception in relation to water, air, and food pollution, and thus is equivalent to EHPI.
- Factor 3 (FCT3) includes only one item that is a measure of road traffic level perception.
- Factor 4 (FCT4) is composed of items evaluating the perception of noise and olfactory pollution (see Table S1 in the Supplementary Material).

Supplementary Figure S1 shows the item loadings associated with the factors where they appeared to be largest in absolute terms. As for the RPI, the Milazzo site presented the highest values for all four factors, which were significantly different among the three sites except for Factor 3 (*p* value = 0.33). 

**Figure 1:**
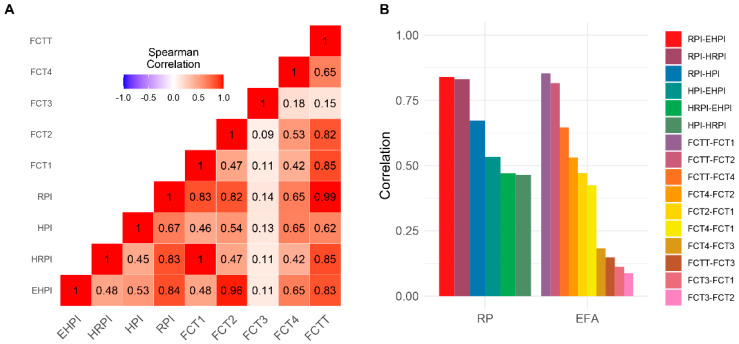
Pairwise correlations between each couple of risk perception (RP) indices and exploratory factorial analysis (EFA) indices. Panel (**A**) shows the Spearman pairwise correlations between each pairing of indices (RP and EFA), whereas Panel (**B**) shows the intra-set correlation values for each set of indices. EHPI: exposure hazard perception index; HRPI: health risk perception index; HPI: hazard perception index; RPI risk perception index. FCT1–4: exploratory factor analysis factors.

The intra-site differences were, instead, all significant for all the indices, except for Factor 3 in the Priolo site and Factor 2 in the Milazzo site. In Crotone, the intra-site differences were not significant (see Table 4). Figure 1, Panel A shows the Spearman pairwise correlations between each pairing of indices (RP and EFA), whereas Panel B shows the intra-set correlation values for each set of indices. The RP set presented a higher mean pairwise-correlation than the EFA set: 0.64 ± 0.17 and 0.43 ± 0.29, respectively.

The total RPI and EFAtot scores were regressed onto “educational level”, NPCSs (the three sites), and “area at risk” (Yes/No). The two indices were significantly associated with all three variables, with the indices increasing with higher educational levels when the area intra-site was at risk with respect to LRAs, and when the sites were Milazzo or Priolo rather than Crotone. The association between the two indices and anxiety status, which was present in 27.6% of the total sample, was studied. The odds were 1.15, close to statistical significance (*p* = 0.06). The same results were obtained with the RPI and EFA indices relevant to health risk perception (HRPI and Factor 1). The model beta coefficient estimates are reported in Supplementary Table S2. Moreover, the indices and health variables were also studied (see Table S3). No significant associations emerged between RP indices and lifestyle variables.

### 3.4. Latent Class Analysis

**Figure 2:**
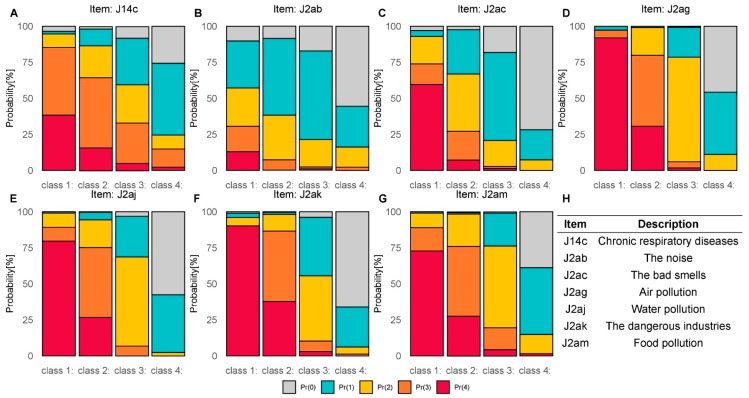
Percentages of responses (from 0 to 4) to the selected items in each class from the latent class analysis (LCA).

The LCA selected seven more information items, as reported in Figure 2. Subjects were then classified into four classes, according to their responses to the selected items. For each class, Figure 2 shows the percentages of responses to each item (from 0 to 4, Likert scale): women in Class 1 were those who felt more exposed to the selected risks, followed by women in Class 2, Class 3, and Class 4.

The RP indices were significantly different among the four classes, increasing as exposure risk perception increased. The same results were obtained for the EFA factors, except for factor 3 (traffic exposure), which was not significantly different in the four classes (Table S4). The NPCS variable (the three sites) was significantly associated with the classification (*p* < 0.001). In the Priolo and Crotone sites, mothers showed a similar distribution profile, with the highest percentages in the intermediate classes (Classes 2 and 3) and the lowest percentages in the extreme classes (Classes 1 and 4). Conversely, inhabitants of the Milazzo site presented a fairly homogeneous distribution in the first three classes, with a very small percentage of women (1.2%) in Class 4, the one with the smallest degree of exposure risk perception. Association between the class variable and area (at-risk versus LRA) was also significant (*p* < 0.001), with similar percentages of women in the first three classes and a low percentage in Class 4 for the at-risk area group. On the contrary, women in the LRAs were distributed more in the intermediate classes, with the lowest percentage in the class at the highest degree of risk exposure perception. Regarding educational level, the percentage of women with the highest degree of qualification and belonging to Class 4 was lower than the percentages in the other three classes (*p* = 0.016) (Table 5).

**Figure 3:**
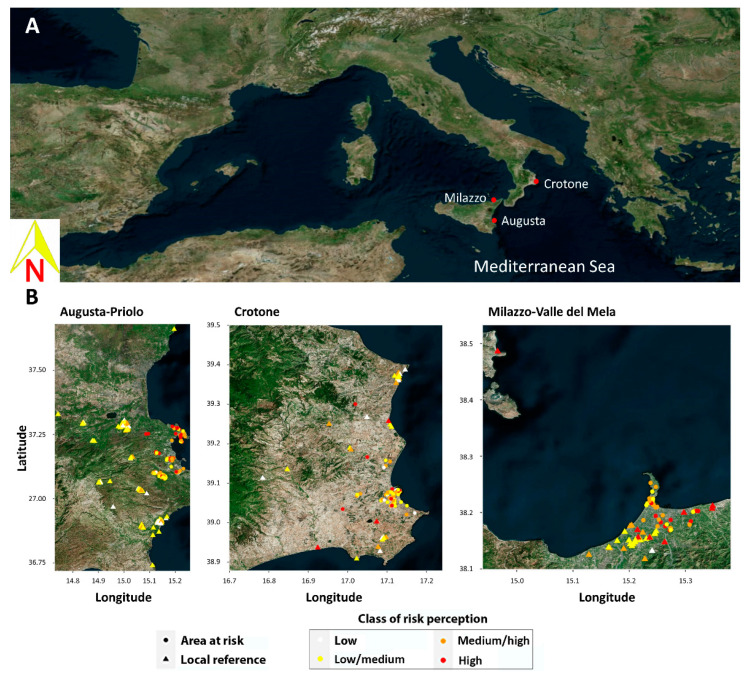
Geographical distribution of mothers in each national priority contaminated site (NPCS). (**A**) The different colours in panel (**B**) are relevant to the different four classes of risk perception (white: low risk; yellow: low/medium; orange: medium/high; red: high). Circles refer to the residence in NPCSs and triangles in local reference areas.

Figure 3 shows the geographical distribution of mothers in each NPCS. The figure shows that, in the Priolo NPCS, most of the mothers living in the at-risk area belonged to the medium/high risk perception classes, while most of the mothers living in the LRA were in the low/medium class. In Crotone, the four classes were homogeneously distributed between the NPCS and LRA areas, while in Milazzo, only one mother perceived a low risk, with the rest equally distributed in the other classes independently from their residence area.

The results of the present work are more extensively presented in the Supplementary Material.

## 4. Discussion

Risk perception during pregnancy is a process influenced by multiple personal, psychological, and societal factors [42]. Pregnant women are increasingly aware that they are experiencing a unique moment in their lives, during which it is necessary to monitor their health conditions and have proper medical management, as well as engage in healthy lifestyle behaviours. All these factors may lead to changes in pregnant women’s perceptions, thus increasing their perception of environmental and health risks [43].

The three studied NPCSs of Milazzo, Priolo, and Crotone have diverse environmental characteristics, with the first two having active refineries and power plants, while the third contains abandoned industrial areas.

In all these contexts, the influence of environmental pollution on pregnant women is of particular interest due to possible exposures to multiple factors recognised as harmful to their health and offspring. Many adverse health impacts of air pollution on birth outcomes and children’s health are well known, while others have not yet been characterised [44], and scientific works have offered recommendations for reducing industrial emissions and calculating costs both for society as a whole as well as for the environment [45].

In the present work, we describe the distribution of lifestyles and health characteristics of a sample including 611 women enrolled at the end of their gestation in the NEHO birth cohort. The women investigated, with an average age of 31.5 ± 4.9 years, had the same characteristics as the 845 total enrolled participants in terms of age, marital status, educational level and work situation [25].

Marital status was not significantly different in the three evaluated sites. Conversely, educational level and work situation were different in the three NPCSs, with the highest educational level in Milazzo as well as the highest percentage of working mothers, as shown in a previously published manuscript [25]. This result is also reflected in the crowding index value which recorded its highest index value in Priolo, the site with the largest number of study participants belonging to the middle and lowest educational levels and having the lowest percentage of working mothers.

To evaluate the RP of pregnant women living in NPCSs and LRAs, four RP indices (HPI, EHPI, HRPI, and RPI) were used. The analyses revealed differences in RP profile for the three sites. The highest values for all indices were observed in the Milazzo site and the lowest in the Crotone site. This result, in line with what has previously been described in the literature, suggests that educational qualifications may increase people’s perceptions of environmental risks. Thus, subjects with a higher level of education perceive environmental risks to be higher [7,46,47]. Furthermore, the lowest RP values found in Crotone could be attributed to the fact that the three industrial plants have been disused since 1990 [32]. Again, in Crotone, we did not find significant differences for all indices between the at-risk areas and LRAs, with the exception of the HPI. On the contrary, RP values were significantly higher in NPCS areas than in LRAs for both the Priolo and Milazzo sites. The perception of the presence of environmental factors with high visual impact (e.g., incinerators, waste-to-energy plants, and wind farms)—as in the case of Milazzo and Priolo—was directly associated with higher RP values when compared to RP reported in areas with less industrial impact [33,37,48,49,50].

In order to test whether additional information from the questionnaire could be useful for the definition of RP, we performed an exploratory factorial analysis, which identified four factors. Among the EFA factors, factor 4 (FCT4), characterised by items evaluating olfactory and noise perception, was of particular interest in the context of industrial areas. Recently, bad smells and noise perception were investigated by an innovative activity of citizen science, NOSE (Network for Odour Sensitivity), a web application allowing citizens to anonymously, in real time and on a georeferenced basis, report bad smells experienced in their surroundings; in particular, in NPCSs in Sicily, this is reported to the Sicilian Regional Agency for Environmental Protection [51]. This activity, as well as RP evaluation, aims at bridging the gap between citizens and institutions, raising awareness, and involving them in environmental monitoring activities. Furthermore, information about the presence of FCT4 could encourage the use of citizen science tools such as NOSE.

The values of the four EFA indices, as for the RP indices, were higher in the Milazzo NPCS. Furthermore, RP and EFA indices were associated with both educational level and NPCSs: in fact, mothers with higher educational levels living in the Milazzo NPCS showed greater attention and concern than mothers with lower educational levels living in Crotone. No significant association was found between each RPI or EFA index and participants’ lifestyles (i.e., maternal smoking habit, physical activity during pregnancy, weight gain, and pregravid BMI—data not shown), indicating that lifestyle behaviours adopted during pregnancy are independent of RP. In interpreting the evidence, it should be considered that the relatively short period of pregnancy might play a limited role in the possibility that RP could bring about changes in previously formed lifestyles.

However, improving lifestyle behaviours adopted during pregnancy, particularly in highly polluted areas, would generate co-benefits by avoiding the addition of further risk factors, such as cigarette smoking and malnutrition, to environmental risks to which foetuses and children are already exposed.

Using an LCA, a selected optimal subset of seven items was identified: chronic respiratory diseases, noise, bad smells, air pollution, water pollution, dangerous industries, and food pollution. The LCA classified participants into four classes, describing four different degrees of RP (high (Class 1), medium/high (Class 2), low/medium (Class 3), and low (Class 4)). The first class was constituted by women who had the highest degree of RP and who felt more exposed to the selected risks, followed by women in Class 2, Class 3, and Class 4, the latter being composed of women with the lowest degree of exposure to the described risks. As shown in Table 5, all the RP and EFA indices were significantly different among the four classes, with values rising with increased value in the exposure RP. Moreover, the results indicated that, among the four different classes identified, NPCS and area variables, educational level, and age influenced mothers’ perception of environmental and health risks, in accordance with the results found for both the RP and EFA indices. This result could be useful for defining the minimum number of items in the RP evaluation, especially in cases of online applications or mobile phone applications developed for surveys specifically aimed at people living in NPCSs. In our opinion, LCA is able to provide a detailed description of RP by characterizing the individual women’s profiles: thus, LCA may constitute a useful basis for a scientific approach to future potential intervention strategies.

Finally, the geographical distribution of the mothers in each NPCS, according to their RP and residence in an at-risk area or LRA, as shown in Figure 3, provided different suggestions depending on the site. In the Priolo NPCS, a net distinction in RP appeared between women living in the at-risk area and those living in the LRA. Similarly, in Milazzo, the nearly complete absence of women with low perceptions of risk highlights that there is a high awareness of the environmental threats that characterise that area. Conversely, in Crotone, the homogeneous distribution of the four classes in the two areas shows that there is no association between environmental risk and the perception of risk.

Remediation interventions often take a long time, which requires policymakers, researchers, and health promotion professionals to encourage healthy lifestyles in residents until the remediation is completed. Thus, the evaluation of RP may be a useful tool to better understand communities living in ICSs, with the aim of providing adequate information to citizens and making people more active in citizen science initiatives [37,52].

## 5. Conclusions

A good understanding of the health of pregnant women living in heavily contaminated areas is fundamental for defining public health strategies. Our analysis provided results and tools for developing ad hoc questionnaires to investigate RP in relation to individual characteristics in a population living in high-risk areas.

For the four classes of RP, the study showed that participants have different RP profiles for health and environmental hazards. Their socioeconomic characteristics, such as education, work situation, and residence in highly industrialised areas, may influence their RP. Furthermore, even if RP could play a role as a health determinant or health effect modifier, in our results, RP was not associated with lifestyles during pregnancy. In a relatively short period of time, similar to that of pregnancy, the perception of environmental risk does not seem to be able to change previously formed lifestyles. Nevertheless, pregnancy is a crucial time window, during which exposure to pollutants and unhealthy lifestyles may result in an increased occurrence of diseases during childhood and adulthood.

We examined the role that RP plays in creating new strategies to improve the active involvement of citizens against environmental pollution, and we were also able to identify a minimum number of selected items to better evaluate RP profiles. LCA allowed us to demonstrate that only seven questions were able to adequately describe RP in pregnant women living in polluted areas.

To our knowledge, to date, no specific studies have been published on the risk perception associated with the living environment and health in mother–child cohorts residing in sites with environmental contamination and designated as remediation sites. We believe our result may be useful for optimizing further online surveys investigating the RP of environmental pollution in highly contaminated areas.

Moreover, it should be emphasised that great attention should be paid to the impact of environmental pollution on children’s health, not only by women living inside NPCSs but also by residents in the surrounding areas in order to enhance awareness of pollution’s impact on children and, in general, on human health. Likely, as previously suggested, the higher RP values in Milazzo and Priolo could also be associated with the presence of operating industrial plants, while in Crotone, the three industrial sites are disused.

Finally, this study on mothers’ RP highlighted the key issues that need to be conveyed in the environmental information provided to citizens through the internet and national/local media.

## Figures and Tables

**Table 1:** Descriptive characteristics of participants to the *Neonatal Environment and Health Outcomes* birth cohort.

	AUGUSTA PRIOLO			CROTONE			MILAZZO			COHORT		
Quantitative Variables	TOTAL	NCPS	LRA	TOTAL	NCPS	LRA	TOTAL	NCPS	LRA	TOTAL	NCPS	LRA
		* *p* value = 0.99			* *p* value = 0.035			* *p* value = 0.14		^&^ *p* value = 0.049	^%^ *p* value = 0.19	
Age (years)	31.3 (±4.9)	31.3 (±4.97)	31.3 (±4.9)	31.2 (±5)	31.8 (±5.3)	29.9 (±4.2)	32.7 (±4.3)	33.3 (±4)	31.8 (±4.6)	31.5 (±4.9)	31.6 (±5)	31.1 (±4.8)
		* *p* value = 0.14			* *p* value = 0.53			* *p* value = 0.33		^&^ *p* value = 0.29	^%^*p* value = 0.07	
BMI (kg/m^2^)	23.5 (±4.6)	23.8 (±4.79)	23.1 (±4.3)	23.2 (±3.8)	23.3 (±4.05)	22.9 (±3.1)	22.7 (±3.8)	23 (±4)	22.2 (±3.6)	23.3 (±4.3)	23.6 (±4.5)	22.9 (±4)
		* *p* value = 0.38			* *p* value = 0.15			* *p* value = 0.61		^&^ *p* value = 0.015	^%^ *p* value = 0.09	
Weight (kg)	12.1 (±4.1)	11.9 (±3.93)	12.3 (±4.3)	11.4 (±4.4)	10.9 (±3.75)	12.3 (±5.2)	13.1 (±4.1)	12.9 (±4.1)	13.4 (±4.2)	12.1 (±4.1)	11.9 (±3.9)	12.5 (±4.5)
		* *p* value = 0.016			* *p* value = 0.23			* *p* value = 0.05		^&^ *p* value = 0.37	^%^ *p* value < 0.01	
Crowding Index	1.16 (±0.56)	1.21 (±0.61)	1.07 (±0.46)	1.13 (±0.54)	1.17 (±0.55)	1.05 (±0.49)	1.06 (±0.45)	1.14 (±0.47)	0.95 (±0.4)	1.14 (±0.54)	1.19 (±0.58)	1.05 (±0.45)

Crowding Index was computed as the total number of co-residents per household divided by the total number of rooms, excluding kitchen and bathrooms. * *p* values from unpaired *t*-test for evaluation of quantitative variable differences between area at risk (NPCS) vs. local reference (LRA) within each site; ^&^ *p* values from ANOVA for evaluation of quantitative variable differences among sites (Augusta–Priolo, Crotone, and Milazzo–Valle del Mela); ^%^ *p* values from unpaired *t*-test for evaluation of quantitative variable differences between area at riskand local reference in the total cohort.

**Table 2:** Descriptive characteristics of participants to the *Neonatal Environment and Health Outcomes* birth cohort: lifestyle and health.

	AUGUSTA PRIOLO			CROTONE			MILAZZO			COHORT		
	TOTAL	NPCS	LRA	TOTAL	NPCS	LRA	TOTAL	NPCS	LRA	TOTAL	NPCS	LRA
	406 (66.5%)	253 (62.3%)	153 (37.7%)	121 (19.8%)	82 (67.8%)	39 (32.2%)	84 (13.7%)	50 (59.5%)	34 (40.5%)	611	385 (63.0%)	226 (37.0%)
Marital status		* *p* value = 0.26			* *p* value = 0.097			* *p* value = 0.65		^&^ *p* value = 0.44	^%^ *p* value = 0.04	
Married	256 (63.4%)	154 (61.3%)	102 (66.7%)	84 (69.4%)	52 (63.5%)	32 (82.1%)	59 (70.2%)	34 (68%)	25 (73.5%)	399 (65.5%)	240 (62.7%)	159 (70.4%)
Maiden	145 (35.9%)	94 (37.5%)	51 (33.3%)	35 (28.9%)	28 (34.1%)	7 (17.9%)	24 (28.6%)	15 (30%)	9 (26.5%)	204 (33.5%)	137 (35.8%)	67 (29.6%)
Divorced/Separated	3 (0.7%)	3 (1.2%)	0 (0%)	2 (1.7%)	2 (2.4%)	0 (0%)	1 (1.2%)	1 (2%)	0 (0%)	6 (1%)	6 (1.5%)	0 (0%)
Total	404 (100%)	251 (100%)	153 (100%)	121 (100%)	82 (100%)	39 (100%)	84 (100%)	50 (100%)	34 (100%)	609 (100%)	383 (100%)	226 (100%)
Educational level		* *p* value = 0.43			* *p* value = 0.67			* *p* value < 0.01		^&^ *p* value = 0.02	^%^ *p* value = 0.13	
Second. school or lower qualific.	74 (18.3%)	50 (19.8%)	24 (15.8%)	17 (14.3%)	10 (12.3%)	7 (18.4%)	5 (6%)	3 (6%)	2 (5.9%)	96 (15.8%)	63 (16.4%)	33 (14.7%)
High school	220 (54.5%)	138 (54.8%)	82 (53.9%)	63 (52.9%)	44 (54.4%)	19 (50%)	44 (52.4%)	33 (66%)	11 (32.4%)	327 (53.9%)	215 (56.2%)	112 (50%)
Degree or higher qualific.	110 (27.2%)	64 (25.4%)	46 (30.3%)	39 (32.8%)	27 (33.3%)	12 (31.6%)	35 (41.6%)	14 (28%)	21 (61.7%)	184 (30.3%)	105 (27.4%)	79 (35.3%)
Total	404 (100%)	252 (100%)	152 (100%)	119 (100%)	81 (100%)	38 (100%)	84 (100%)	50 (100%)	34 (100%)	607 (100%)	383 (100%)	224 (100%)
Working condition		* *p* value = 0.44			* *p* value = 0.014			* *p* value = 0.09		^&^ *p* value < 0.01	^%^ *p* value = 0.85	
NO	267 (66.6%)	170 (68%)	97 (64.2%)	62 (52.1%)	36 (44.4%)	26 (68.4%)	26 (31%)	19 (38%)	7 (20.6%)	355 (58.8%)	225 (59.1%)	130 (58.3%)
YES	134 (33.4%)	80 (32%)	54 (35.8%)	57 (47.9%)	45 (55.6%)	12 (31.6%)	58 (69%)	31 (62%)	27 (79.4%)	249 (41.2%)	156 (40.9%)	93 (41.7%)
Total	401 (100%)	250 (100%)	151 (100%)	119 (100%)	81 (100%)	38 (100%)	84 (100%)	50 (100%)	34 (100%)	604 (100%)	381 (100%)	223 (100%)
Active smoke		* *p* value = 0.57			* *p* value = 0.96			* *p* value = 0.34		^&^ *p* value = 0.53	^%^ *p* value = 0.84	
NO	366 (90.6%)	229 (91.2%)	137 (89.5%)	111 (92.5%)	75 (92.6%)	36 (92.3%)	79 (94%)	46 (92%)	33 (97.1%)	556 (91.4%)	350 (91.6%)	206 (91.2%)
YES	38 (9.4%)	22 (8.8%)	16 (10.5%)	9 (7.5%)	6 (7.4%)	3 (7.7%)	5 (6%)	4 (8%)	1 (2.9%)	52 (8.6%)	32 (8.4%)	20 (8.8%)
Total	404 (100%)	251 (100%)	153 (100%)	120 (100%)	81 (100%)	39 (100%)	84 (100%)	50 (100%)	34 (100%)	608 (100%)	382 (100%)	226 (100%)
Passive smoke		* *p* value = 0.10			* *p* value = 0.93			* *p* value = 0.87		^&^ *p* value < 0.01	^%^ *p* value = 0.24	
NO	377 (92.9%)	239 (94.5%)	138 (90.2%)	105 (86.8%)	71 (86.6%)	34 (87.2%)	65 (77.4%)	39 (78%)	26 (76.5%)	547 (89.5%)	349 (90.6%)	198 (87.6%)
YES	29 (7.1%)	14 (5.5%)	15 (9.8%)	16 (13.2%)	11 (13.4%)	5 (12.8%)	19 (22.6%)	11 (22%)	8 (23.5%)	64 (10.5%)	36 (9.4%)	28 (12.4%)
Total	406 (100%)	253 (100%)	153 (100%)	121 (100%)	82 (100%)	39 (100%)	84 (100%)	50 (100%)	34 (100%)	611 (100%)	385 (100%)	226 (100%)
Consumption of alcohol		* *p* value = 0.41			* *p* value = 0.10			* *p* value = 0.24		^&^ *p* value < 0.01	^%^ *p* value = 0.04	
NO	400 (98.8%)	248 (98.4%)	152 (99.3%)	107 (91.5%)	69 (88.5%)	38 (97.4%)	72 (85.7%)	41 (82%)	31 (91.2%)	579 (95.5%)	358 (94.2%)	221 (97.8%)
YES	5 (1.2%)	4 (1.6%)	1 (0.7%)	10 (8.5%)	9 (11.5%)	1 (2.6%)	12 (14.3%)	9 (18%)	3 (8.8%)	27 (4.5%)	22 (5.8%)	5 (2.2%)
Total	405 (100%)	252 (100%)	153 (100%)	117 (100%)	78 (100%)	39 (100%)	84 (100%)	50 (100%)	34 (100%)	606 (100%)	380 (100%)	226 (100%)
Sport		* *p* value = 0.18			* *p* value = 0.21			* *p* value = 0.09		^&^ *p* value < 0.01	^%^ *p* value = 0.34	
NO	385 (94.8%)	237 (93.7%)	148 (96.7%)	105 (86.8%)	69 (84.1%)	36 (92.3%)	75 (89.3%)	47 (94%)	28 (82.4%)	565 (92.5%)	353 (91.7%)	212 (93.8%)
YES	21 (5.2%)	16 (6.3%)	5 (3.3%)	16 (13.2%)	13 (15.9%)	3 (7.7%)	9 (10.7%)	3 (6%)	6 (17.6%)	46 (7.5%)	32 (8.3%)	14 (6.2%)
Total	406 (100%)	253 (100%)	153 (100%)	121 (100%)	82 (100%)	39 (100%)	84 (100%)	50 (100%)	34 (100%)	611 (100%)	385 (100%)	226 (100%)
Pressure		* *p* value = 0.51			* *p* value = 0.06			* *p* value = 0.35		^&^ *p* value = 0.55	^%^ *p* value = 0.34	
NO	366 (90.1%)	230 (90.9%)	136 (88.9%)	113 (93.4%)	79 (96.3%)	34 (87.2%)	76 (90.5%)	44 (88%)	32 (94.1%)	555 (90.8%)	353 (91.7%)	202 (89.4%)
YES	40 (9.9%)	23 (9.1%)	17 (11.1%)	8 (6.6%)	3 (3.7%)	5 (12.8%)	8 (9.5%)	6 (12%)	2 (5.9%)	56 (9.2%)	32 (8.3%)	24 (10.6%)
Total	406 (100%)	253 (100%)	153 (100%)	121 (100%)	82 (100%)	39 (100%)	84 (100%)	50 (100%)	34 (100%)	611 (100%)	385 (100%)	226 (100%)
Cardiovascular		* *p* value = 0.81			-			* *p* value = 0.42		^&^ *p* value = 0.41	^%^ *p* value = 0.64	
NO	390 (98.5%)	241 (98.4%)	149 (98.7%)	118 (100%)	80 (100%)	38 (100%)	81 (98.8%)	49 (98%)	32 (100%)	589 (98.8%)	370 (98.7%)	219 (99.1%)
YES	6 (1.5%)	4 (1.6%)	2 (1.3%)				1 (1.2%)	1 (2%)	0 (0%)	7 (1.2%)	5 (1.3%)	2 (0.9%)
Total	396 (100%)	245 (100%)	151 (100%)	118 (100%)	80 (100%)	38 (100%)	82 (100%)	50 (100%)	32 (100%)	596 (100%)	375 (100%)	221 (100%)
Cholesterol		* *p* value = 0.11			*p value = 0.40			* *p* value = 0.54		^&^ *p* value = 0.96	^%^ *p* value = 0.45	
NO	374 (94.4%)	233 (95.9%)	141 (92.2%)	112 (94.9%)	75 (93.8%)	37 (97.4%)	77 (95.1%)	46 (93.9%)	31 (96.9%)	563 (94.6%)	354 (95.2%)	209 (93.7%)
YES	22 (5.6%)	10 (4.1%)	12 (7.8%)	6 (5.1%)	5 (6.3%)	1 (2.6%)	4 (4.9%)	3 (6.1%)	1 (3.1%)	32 (5.4%)	18 (4.8%)	14 (6.3%)
Total	396 (100%)	243 (100%)	153 (100%)	118 (100%)	80 (100%)	38 (100%)	81 (100%)	49 (100%)	32 (100%)	595 (100%)	372 (100%)	223 (100%)
Psychic disorders		* *p* value = 0.72			* *p* value = 0.38			* *p* value = 0.64		^&^ *p* value = 0.54	^%^ *p* value = 0.81	
NO	285 (70.2%)	176 (69.6%)	109 (71.2%)	87 (71.9%)	61 (74.4%)	26 (66.7%)	64 (76.2%)	39 (78%)	25 (73.5%)	436 (71.4%)	276 (71.7%)	160 (70.8%)
YES	121 (29.8%)	77 (30.4%)	44 (28.8%)	34 (28.1%)	21 (25.6%)	13 (33.3%)	20 (23.8%)	11 (22%)	9 (26.5%)	175 (28.6%)	109 (28.3%)	66 (29.2%)
Total	406 (100%)	253 (100%)	153 (100%)	121 (100%)	82 (100%)	39 (100%)	84 (100%)	50 (100%)	34 (100%)	611 (100%)	385 (100%)	226 (100%)

* *p* values from a chi-squared test for association between the qualitative variables and the area variable (area at risk (NPCS) vs. local reference (LRA)) within each site; ^&^ *p* values from a chi-squared test for association between the qualitative variables and the site variable (Augusta–Priolo, Crotone, and Milazzo–Valle del Mela); ^%^ *p* values from a chi-squared test for association between the qualitative variable and the area variable (area at risk vs. local reference) in the total cohort.

**Table 3:** Descriptive characteristics of risk perception indices between the three national priority contaminated sites.

	AUGUSTA PRIOLO			CROTONE			MILAZZO			COHORT		
Indexes	TOTAL	NPCS	LRA	TOTAL	NPCS	LRA	TOTAL	NPCS	LRA	TOTAL	NPCS	LRA
		* *p* value < 0.001			* *p* value = 0.16			* *p* value = 0.03		^&^ *p* value < 0.001	^%^ *p* value < 0.001	
EHPI	0.53 (±0.23)	0.59 (±0.22)	0.44 (±0.21)	0.47 (±0.24)	0.49 (±0.23)	0.43 (±0.26)	0.61 (±0.2)	0.65 (±0.18)	0.56 (±0.21)	0.53 (±0.23)	0.58 (±0.22)	0.45 (±0.22)
		* *p* value < 0.001			* *p* value = 0.82			* *p* value = 0.01		^&^ *p* value < 0.01	^%^ *p* value < 0.001	
HRPI	0.58 (±0.27)	0.63 (±0.27)	0.51 (±0.26)	0.58 (±0.24)	0.58 (±0.24)	0.59 (±0.24)	0.68 (±0.2)	0.73 (±0.15)	0.59 (±0.23)	0.59 (±0.26)	0.63 (±0.25)	0.53 (±0.26)
		* *p* value < 0.001			* *p* value < 0.01			* *p* value = 0.04		^&^ *p* value < 0.001	^%^ *p* value < 0.001	
HPI	0.45 (±0.26)	0.51 (±0.26)	0.33 (±0.23)	0.34 (±0.23)	0.38 (±0.23)	0.25 (±0.18)	0.57 (±0.24)	0.61 (±0.22)	0.5 (±0.24)	0.44 (±0.26)	0.5 (±0.26)	0.35 (±0.23)
		* *p* value < 0.001			* *p* value = 0.45			* *p* value < 0.01		^&^ *p* value < 0.001	^%^ *p* value < 0.001	
RPI	0.54 (±0.22)	0.6 (±0.21)	0.45 (±0.19)	0.5 (±0.18)	0.51 (±0.18)	0.48 (±0.19)	0.64 (±0.16)	0.69 (±0.13)	0.57 (±0.18)	0.55 (±0.21)	0.59 (±0.2)	0.47 (±0.19)

* *p* values from unpaired *t*-test for evaluation of index differences between area at risk (NPCS) vs. local reference (LRA) within each site; ^&^ *p* values from ANOVA for evaluation of index differences among sites (Augusta–Priolo, Crotone, and Milazzo–Valle del Mela); ^%^ *p* values from unpaired *t*-test for evaluation of index differences between area at risk vs. local reference in the total cohort. EHPI: exposure hazard perception index; HRPI: health risk perception index; HPI: hazard perception index; RPI: risk perception index.

**Table 4:** Risk perception indices from the exploratory factor analysis (EFA).

	AUGUSTA PRIOLO			CROTONE			MILAZZO			COHORT		
Indexes	TOTAL	NPCS	LRA	TOTAL	NPCS	LRA	TOTAL	NPCS	LRA	TOTAL	NPCS	LRA
		* *p* value < 0.001			* *p* value = 0.83			* *p* value < 0.01		^&^ *p* value < 0.01	^%^ *p* value < 0.01	
FACTOR 1	0.58 (±0.27)	0.63 (±0.27)	0.5 (±0.26)	0.57 (±0.24)	0.57 (±0.24)	0.58 (±0.24)	0.68 (±0.2)	0.73 (±0.15)	0.59 (±0.23)	0.59 (±0.26)	0.63 (±0.25)	0.53 (±0.26)
		* *p* value < 0.001			* *p* value = 0.11			* *p* value = 0.09		^&^ *p* value < 0.001	^%^ *p* value < 0.01	
FACTOR 2	0.59 (±0.25)	0.64 (±0.25)	0.49 (±0.24)	0.54 (±0.27)	0.57 (±0.25)	0.47 (±0.31)	0.68 (±0.22)	0.71 (±0.21)	0.63 (±0.22)	0.59 (±0.26)	0.64 (±0.25)	0.51 (±0.25)
		* *p* value = 0.68			* *p* value = 0.09			* *p* value = 0.01		^&^*p* = 0.33	^%^ *p* value = 0.03	
FACTOR 3	0.47 (±0.26)	0.48 (±0.26)	0.46 (±0.27)	0.46 (±0.34)	0.49 (±0.32)	0.37 (±0.37)	0.51 (±0.3)	0.58 (±0.28)	0.41 (±0.3)	0.47 (±0.28)	0.49 (±0.28)	0.44 (±0.29)
		* *p* value < 0.001			* *p* value = 0.48			* *p* value < 0.001		^&^ *p* value < 0.001	^%^ *p* value < 0.01	
FACTOR 4	0.44 (±0.21)	0.5 (±0.21)	0.34 (±0.17)	0.32 (±0.19)	0.33 (±0.2)	0.31 (±0.18)	0.49 (±0.22)	0.56 (±0.18)	0.39 (±0.23)	0.42 (±0.22)	0.47 (±0.22)	0.34 (±0.18)
		* *p* value < 0.001			* *p* value = 0.62			* *p* value < 0.001		^&^ *p* value < 0.001	^%^ *p* value < 0.01	
TOTAL EFA	0.56 (±0.22)	0.62 (±0.21)	0.47 (±0.19)	0.52 (±0.19)	0.53 (±0.19)	0.51 (±0.19)	0.65 (±0.16)	0.71 (±0.13)	0.58 (±0.18)	0.57 (±0.21)	0.61 (±0.21)	0.49 (±0.19)

* *p* values from unpaired *t*-test for evaluation of index differences between area at risk vs. local reference within each site; ^&^ *p* values from ANOVA for evaluation of index differences among sites (Augusta–Priolo, Crotone, and Milazzo–Valle del Mela); ^%^ *p* values from unpaired *t*-test for evaluation of index differences between area at risk (NPCS) vs. local reference (LRA) in the total cohort.

**Table 5:** Percentages or average mean and standard deviation of the national priority contaminated sites, area, educational level, and age, according to the classes from the latent class analysis (LCA).

	CLASS 1 High Risk	CLASS 2 Medium/High	CLASS 3 Low/Medium	CLASS 4 Low Risk		TOTAL
COHORT	105 (17.2%)	209 (34.2%)	224 (36.7%)	73 (11.9%)	*p* value	611
NPCS						
AUGUSTA-PRIOLO	68 (16.7%)	141 (34.7%)	148 (36.5%)	49 (12.1%)	** *p* value < 0.001	406 (66.4%)
CROTONE	14 (11.6%)	35 (28.9%)	49 (40.5%)	23 (19%)		121 (19.8%)
MILAZZO-VALLE del MELA	23 (27.4%)	33 (39.3%)	27 (32.1%)	1 (1.2%)		84 (13.7%)
AREA						
AT RISK AREA	91 (23.6%)	143 (37.1%)	120 (31.2%)	31 (8.1%)	** *p* value < 0.001	385 (63%)
LOCAL REFERENCE AREA	14 (6.2%)	66 (29.2%)	104 (46%)	42 (18.6%)		226 (37%)
Educational level						
secondary school or lower qualification	20 (20.8%)	27 (28.1%)	30 (31.3%)	19 (19.8%)	** *p* value = 0.016	96 (15.8%)
high school	54 (16.5%)	122 (37.4%)	111 (33.9%)	40 (12.2%)		327 (53.9%)
Degree or higher qualification	30 (16.3%)	58 (31.5%)	82 (44.6%)	14 (7.6%)		184 (30.3%)
Total	104 (17.1%)	207 (34.1%)	223 (36.8%)	73 (12%)		607 (100%)
AGE	31.8 ±(4.99)	32 ±(4.71)	31.3 ±(4.79)	30.1 ±(5.33)	* *p* value = 0.03	31.5 ±(4.89)

*p* value for differences among classes; * one-way ANOVA; ** chi-squared test.

## Data Availability

No applicable.

## Supplementary Materials

The following are available online at https://www.mdpi.com/article/10.3390/ijerph182010616/s1, 1. Supplementary methods: 1.1 Questionnaire on Risk Perception, 1.2 Risk Perception indices, 1.3 Exploratory Factorial Analysis indices, 1.4 Latent Class Analysis. 2. Supplementary Results: 2.1 Socio-demographic information, 2.2 Risk perception indices, 2.3 Exploratory Factorial Analysis indices, 2.4 Latent Class Analysis. Table S1: RP Item Description, Table S2. Results from the beta-regression model, Table S3: Average mean and standard deviation of the RP indices and EFA indices according to the “medical variables”, Table S4. Average mean and standard deviation of the Risk Perception and Exploratory Factor Analysis indices, according to the classes from the Latent Class Analysis. Figure S1: Item Loadings from the Exploratory Factorial Analysis.
