# Supplementary material for: Measuring Risk Perception in Pregnant Women in Heavily Polluted Areas: A New Methodological Approach from the NEHO Birth Cohort
Source: Int J Environ Res Public Health. 2021 Oct 11;18(20):10616. doi: 10.3390/ijerph182010616 (PMC8536087; doi:10.3390/ijerph182010616)
Supplement: Supplementary file 1 [file ijerph-18-10616-s001.zip › ijerph-1403970-supplementary.pdf]

## 1. Supplementary methods

Between January 2018 and January 2020, the NEHO cohort enrolled, on a voluntary basis, 845 pregnant women living in the 3 NPCSs of Crotone, Priolo and Milazzo in the Mediterranean area of Southern Italy, along with pregnant women living in surrounding areas (Local Reference Areas, LRAs), outside the perimeter of the relevant NPCSs but presenting similar geographic and socio-demographic characteristics (Ruggieri et al., 2021). Women living in the LRAs represent the control group. The study is being conducted following the Declaration of Helsinki. All the adopted procedures comply with the General Data Protection Regulation (UE 2016/679) and Italian laws concerning data protection.

### *1.1 Questionnaire on Risk Perception*

After enrolment in the NEHO cohort study, mothers were asked to fill out a questionnaire collecting information on maternal health and lifestyle during the gestational and pre-gestational periods. Information on the risk perception of pregnant women was also collected by means of a questionnaire used in several previous human biomonitoring surveys (Signorino and Beck, 2014, Coi et al., 2016, Bena et al., 2019, Dettori et al., 2020).

In the present work, we analysed a subset of questions from the J section of this questionnaire, composed of 14 questions about risk perception requiring answers on a Likert scale (from 0 to 4) or expressing the "presence" or "absence" of a certain risk. Some of these questions were used to compute Risk Perception (RP) indices (the selected questions are reported in supplementary Table S1 and indicated with the letter J). These questions examined the perception of being exposed to air, water and food pollution, as well as to smells, noise and the presence of dangerous industries. The perception of the presence of diseases related to environmental pollution, such as infertility, chronic respiratory disease, allergies and various types of cancer, was also explored. In the process of evaluating the risk perception of exposure, three additional questions were analysed to evaluate the presence of vehicular traffic and its relative impact on air quality (indicated with the letter E in Table S1).

**Supplementary materials Table S1**. RP item Description

| Item | **Description** | **Risk Perception Index** | **EFA Index** |
|---|---|---|---|
| E13 | *Your home is located in an area: (without traffic-with little traffic-with moderate traffic-with heavy traffic)* | | FACTOR3 |
| E16 | *On the street where you live, how often do trucks drive by on working days?(Never or almost never-Every now and then –Frequently – Continuously)* | | FACTOR4 |
| E22 | *How much annoyance does pollution give you, e.g. Does it smell like smog / exhaust fumes when you keep the windows open at home?(0=not at all; 10=a lot)* | | FACTOR4 |
| J2 | ***To what extent do you feel personally exposed to: (Not at all-a little-enough-very-very much)*** | | |
| J2ab | *Noise* | EHPI | FACTOR4 |
| J2ac | *Bad smells* | EHPI | FACTOR4 |
| J2ag | *Air pollution* | EHPI | FACTOR2 |
| J2aj | *Water pollution* | EHPI | FACTOR2 |
| J2ak | *Dangerous industries* | EHPI | FACTOR2 |
| J2al | *Earthquakes* | EHPI | FACTOR2 |
| J2am | *Food pollution* | EHPI | FACTOR2 |
| J3 | ***Among the dangers listed above, which do you think are present in the area where you live? (NO-YES)*** | | |
| J3b | *Noise* | HPI | FACTOR4 |
| J3c | *Bad smells* | HPI | FACTOR4 |
| J3g | *Air pollution* | HPI | FACTOR4 |
| J3j | *Water pollution* | HPI | FACTOR2 |
| J3k | *Dangerous industries* | HPI | FACTOR4 |
| J3l | *Earthquakes* | HPI | FACTOR2 |
| J3m | *Food pollution* | HPI | FACTOR2 |
| J14 | ***In your opinion, how likely is it, in your area of residence, to have: (Not at all probable-Unlikely-Medium probable-Very probable-Certain)*** | | |
| J14a | *Allergies* | HRPI | FACTOR1 |
| J14b | *Acute respiratory diseases* | HRPI | FACTOR1 |
| J14c | *Chronic respiratory diseases* | HRPI | FACTOR1 |
| J14d | *Cardiovascular diseases* | HRPI | FACTOR1 |
| J14e | *Infertility* | HRPI | FACTOR1 |
| J14f | *Various forms of cancer* | HRPI | FACTOR1 |
| J14g | *Leukaemia* | HRPI | FACTOR1 |
| J14h | *Congenital malformations* | HRPI | FACTOR1 |

EHPI: Exposure Hazard Perception Index; HRPI: the Health Risk Perception Index; HPI: Hazard Perception Index.
FACTOR 1-4: Exploratory Factor Analysis factors

*1.2 Risk Perception indices*

The questions included in the J section of the questionnaire were used to compute the four RP indices according to the indications and formulas reported in previous works (Signorino and Beck, 2014, Minichilli et al., 2018, Bianchi et al., 2019). A total number of 22 items were involved in constructing the indices. The original formulation was modified to obtain a value for each enrolled mother.

The computed RP indices were:

- the Hazard Perception Index (HPI);
- the Exposure Hazard Perception Index (EHPI);
- the Health Risk Perception Index (HRPI);
- the Risk Perception Index (RPI).

HPI indicated the mothers' perceptions of the presence or absence of certain hazards in their residence area. EHPI reflected questions about perceptions that mothers have of being exposed to a certain danger in the area where they live. HRPI investigated the perception of health risks, whether in the participant's opinion certain diseases such as allergies, chronic respiratory diseases, infertility and various types of cancer could arise in the area where they live. Finally, RPI included the overall perception of both environmental and health risks, taking the different perceptions described above together (Table S1).

For each participant, the HPI, EHPI, HRPI, and RPI were calculated as follows:

$$I = \frac{1}{K \times J}\left(\sum_{i=1}^{K} n_i x_i\right)$$

where $x_i$ is the i-th modality of the j-th item (j = 1, ..., J) that enters in the computation of the I index; $n_i$ is the number of times that the modality i (i = 1, ..., K) was chosen by the responder in the items used for the computation of the I index, with K being the maximum score on a Likert scale attributable to each item. In this way, each index takes a value from 0 to 1.

The Spearman rank correlation test was used to check for possible pairwise correlations between each pair of indices.

*1.3 Exploratory Factorial Analysis indices*

Aimed at verifying whether additional information regarding risk perception definition would be better able to discriminate between participants from contaminated sites and LRAs, an Exploratory Factorial Analysis (EFA) involving a larger number of items, for a total of 28, was used to automatically define a set of four "latent factors" (indices), dependent on the included items. A global index of risk perception, EFAtot, was defined as the sum of the four factors.

To evaluate the "factorability" of the data, the Kaiser-Meyer-Olkin test (KMO) and Bartlett's test were computed. The KMO test measures sampling adequacy (sample of included variables) in terms of variable correlations. Higher values indicate that a factor analysis can be performed; conversely, a value of less than 0.60 indicates that the sample is not adequate. Bartlett's sphericity test was used on the correlation matrix to determine if the included items were unrelated, giving insight into the appropriateness of the procedure. A P value of less than 0.05 indicated that a factor analysis could be carried out with the present data.

The number of factors was chosen by means of Cattell's scree plot, i.e., by means of the values assumed by the eigenvalues related to the built factors.

A maximum likelihood approach was used to estimate the factorial loadings, and an oblique rotation method (PROMAX) was used to take correlation among factors into account. The goodness of fit of the factor analysis was evaluated by means of Root Mean Squared Error of Approximation (RMSEA) and the Tucker Lewis Index (TLI). An RMSEA value lower than 0.05 indicates a minimal approximation error; values in the range 0.05-0.08 indicate an acceptable approximation error; values greater than 0.08 indicate a large approximation error with a poor model fitting. A very good TLI is obtained for values

greater than 0.95; good results are obtained for values of the index between 0.9 and 0.95; values less than 0.9 and 0.8 indicate medium and bad performances, respectively. Each factor was built by considering only the items for which the item loadings associated with the factor were the largest in absolute terms. For each mother, the factor value was computed by weighting her responses to the items, constituting the factor, with the item loadings. All the values were normalized to vary between 0 and 1.

Continuous variables are reported as mean ± SD, while categorical variables are presented as numbers and percentages. For continuous variables and the computed indices, one-way ANOVA was used to test for possible differences among women living in the three NPCSs of Priolo, Milazzo and Crotone. A t-test and a Wilcoxon test were used to test differences between at-risk areas and LRAs. A chi-squared test or a Fisher's exact test, when appropriate, was used to study the association between a "site" (NPCS) and categorical variables.

Beta regression models were employed to evaluate the relationships between indices and quantitative or qualitative predictors. The class of beta regression models is a generalization of the logit models in case the response is continuous in the interval (0,1), based on the assumption that the response is beta distributed (Ferrari and Cribari-Neto, 2004). In order to evaluate whether higher perceived risks influence healthier behaviour, lifestyle variables (such as smoking, alcohol consumption, physical activity, weight gain and BMI) were evaluated in a logistic or linear model with each of the computed indices as dependent variables.

### 1.4 Latent Class Analysis

Latent Class Analysis (LCA) was used to discover underlying response patterns and groups of respondents with similar characteristics, identifying the questionnaire items which i) best described risk perception in highly industrialized areas, and ii) in this context, allowed them to be characterized from a geographical and socio-demographic perspective. We selected an optimal subset of items, excluding redundant and non-informative variables for subject classification. The construction of latent class clusters is achieved by maximizing the log-likelihood, and the Expectation-Maximization algorithm is used for optimization. The optimal number of latent classes was chosen through an iterative process based on the Akaike information criterion and the Bayesian information criterion. Furthermore, following the hypothesis that risk perception could play a role as a health determinant or health effect modifier, we evaluated whether pregnant women with higher perceived risks adopted healthier behaviours than those with lower risk perception.

All the analyses were performed in R (R Core Team, 2020).

## 2. Supplementary Results

### 2.1 Socio-demographic information

Out of the 845 women who enrolled in the NEHO cohort, 713 completed the baseline questionnaires, collecting information on maternal health and lifestyle during the pre-gestational period. Of these, 611 women (406 [66.5%] from the Priolo site, 121 [19.8%] from Crotone, and 84 [13.7%] from Milazzo) answered the questions in the risk perception J section and were included in the present study.

Table 1 and Table 2 report relevant quantitative and qualitative characteristics, respectively, of the sample of mothers included in the analyses. Mean age was 31.5±4.9 yrs, and the difference among the three sites was borderline (P=0.049), with slightly older mothers in the Milazzo site. Marital status (married, never-married, divorced/separated) was not significantly different (P=0.44), mimicking the distribution of the total sample, with the largest percentage being married women (65.5%) followed by never-married (33.5%) and divorced/separated women (1%).

As shown in Table 2, the distribution of educational level was different in the three sites (P=0.02): we found the largest percentage of graduated mothers in Milazzo, 41.6%,

versus 32.8% and 27.2% in Crotone and Priolo, respectively. Moreover, in the Milazzo area, there was a significant difference between the NPCS and the LRA, with a larger percentage of women with higher educational levels in the LRA (28% vs 61.7%, $P<0.01$).

The crowding index reflects the educational level, even though the index was not significantly different among the three sites ($P=0.37$): the highest value was recorded in Priolo (1.16±0.56), followed by Crotone (1.13±0.54) and Milazzo (1.06±0.45).

### 2.2 Risk perception indices

Average values of the RP indices HPI, EHPI, HRPI, and RPI, are reported in Table 3. They were all significantly different among the three sites, with the highest values for all the indices observed in Milazzo. The lowest values were observed in Crotone (see Table 3). While within the Priolo and Milazzo sites there was a significant difference between at-risk areas and LRAs, in Crotone only the HPI turned out to be significantly differentiated between the two types of areas (0.38±0.23 and 0.25±0.18 in the at-risk area and the LRA, respectively, $P<0.01$).

### 2.3 Exploratory Factorial Analysis indices

The EFA highlighted the presence of four "latent factors" (indices). The KMO test (0.91) and Bartlett's test ($P<0.05$) indicated the appropriateness of the EFA. The RMSEA and TLI were 0.08 and 0.9, showing a good adaptation of the model to data.

- Factor 1 (FCT1) coincides, in term of building items, with the HRPI index, evaluating the health aspects of the perceived risk.
- Factor 2 (FCT2) includes the items measuring the degree of risk perception in relation to water, air and food pollution, assuming therefore the meaning of EHPI.
- Factor 3 (FCT3) includes only one item that is a measure of road traffic level perception.
- Factor 4 (FCT4) is composed of items evaluating the perception of noise and olfactory pollution (see Table S1 in the supplementary material).

Supplementary Figure S1 shows the item loadings associated with the factors where they appeared to be largest in absolute terms. A global index of risk perception, EFAtot, was also computed as the sum of the four factors. As for the RPI, the Milazzo site presented the highest values for all four factors, which were significantly different among the three sites except for Factor 3 (P value=0.33) whose average trend, however, reflected the trend of all other indices, recording the highest value in Milazzo.

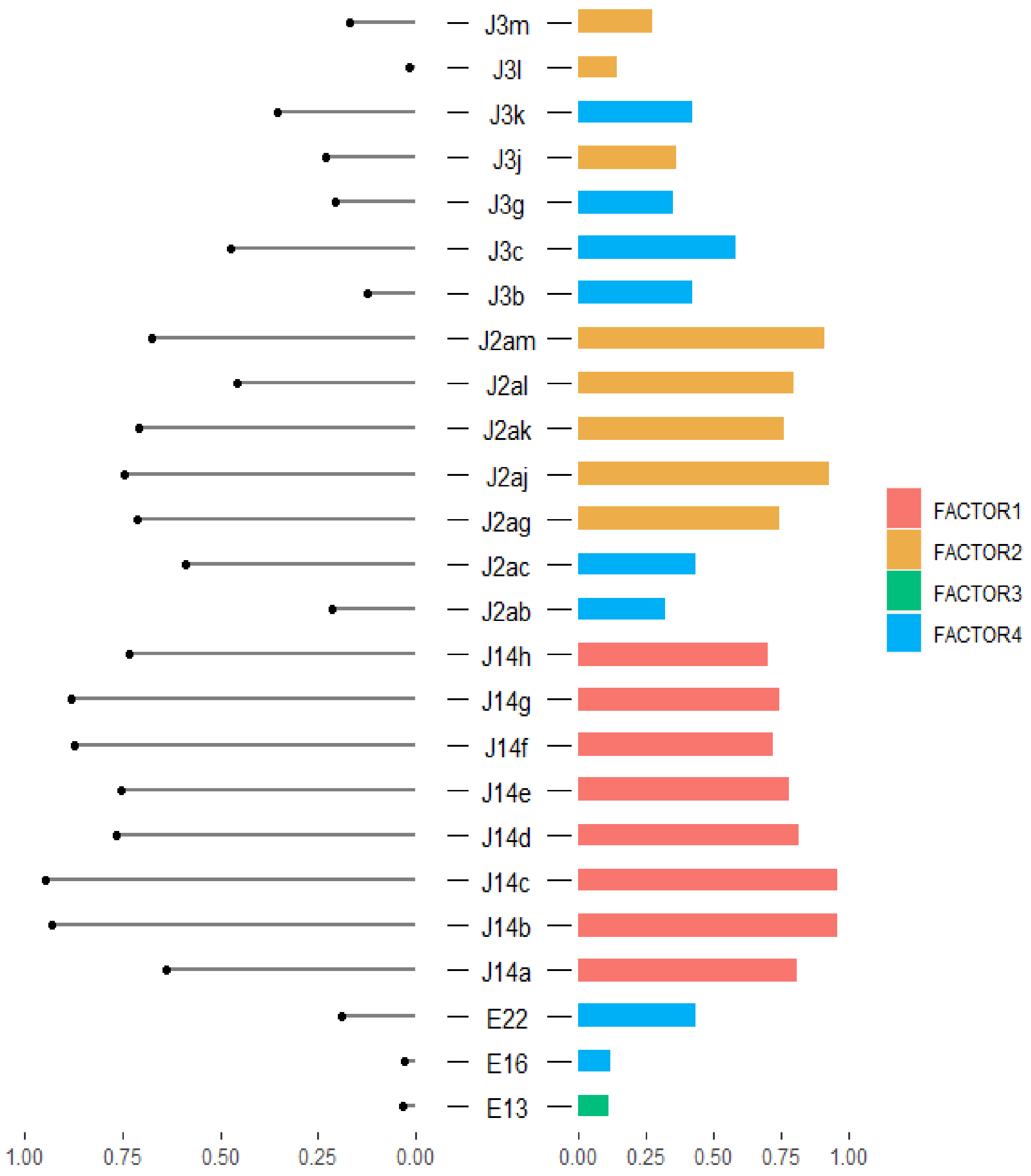


**Figure 1.** Item Loadings from the Exploratory Factorial Analysis. (Refer to Table S1 for item description). For each variable, Communality indicates the fraction of the variable's variance explained by the factorial analysis; Loadings represent the strength of the relationship of each variable with the extracted factors.

The intra-site differences were, instead, all significant for all the indices except for Factor 3 in the Priolo site and Factor 2 in the Milazzo site, where the P values were 0.68 and 0.09, respectively. In Crotone, the intra-site differences were not significantly distinct, with all P values greater than 0.05 (see Table 4). Figure 1, Panel A, shows the Spearman's pairwise correlations between each pairing of indices (RP and EFA), whereas Panel B shows the intra-set correlation values for each set of indices. The RP set presented a higher mean pairwise-correlation than the EFA set: 0.64±0.17 and 0.43±0.29, respectively.

The total RPI and EFAtot scores were regressed onto "educational level", NPCSs (the three sites), and "area at risk" (Yes/No). The two indices were significantly associated with all three variables, with the indices increasing with higher educational levels when the intra-site area was at risk with respect to LRAs and when the sites were Milazzo or Priolo rather than Crotone. The association between the two indices and anxiety status, which was present in 27.6% of the total sample, was studied. The odds were 1.15, close to statistical significance (P=0.06). The same results were obtained with the RPI and EFA indices relevant to health risk perception (HRPI and Factor 1). The model beta coefficient estimates are reported in supplementary Table S2. Moreover, the indices and health variables were also studied (see Table S3). No significant associations emerged between RP indices and lifestyle variables.

**Supplementary materials Table S2**. Results from the beta-regression model.

| | RP indices | | | | EFA indices | | | |
|---|---|---|---|---|---|---|---|---|
| | **estimate** | **exp** | **std.error** | **p.value** | **estimate** | **exp** | **std.error** | **p.value** |
| (Intercept) | 0.08 | 1.09 | 0.09 | 0.33 | 0.20 | 1.22 | 0.09 | 0.02 |
| High school | 0.29 | 1.34 | 0.09 | 0.001 | 0.27 | 1.30 | 0.09 | <0.01 |
| Degree or higher qualification | 0.38 | 1.46 | 0.10 | <0.001 | 0.32 | 1.38 | 0.10 | <0.01 |
| NPCS_CRO | -0.23 | 0.79 | 0.08 | 0.01 | -0.22 | 0.80 | 0.08 | <0.01 |
| NPCS_MIL | 0.37 | 1.45 | 0.10 | <0.001 | 0.33 | 1.39 | 0.10 | <0.01 |
| Local_reference | -0.48 | 0.62 | 0.07 | <0.001 | -0.48 | 0.62 | 0.07 | <0.001 |

**Reference levels for the model:** Educational level: secondary school or lower qualification; NPCS: Priolo; Area: area at risk.

**Supplementary materials Table S3**: Average mean and standard deviation of the RP indices and EFA indices according to the "medical variables".

| INDEX | | N | EHPI | HRPI | HPI | RPI | FACTOR 1 | FACTOR 2 | FACTOR 3 | FACTOR 4 | EFA_TOT |
|---|---|---|---|---|---|---|---|---|---|---|---|
| Pressure | | | 0.29 | 0.52 | 0.1 | 0.26 | 0.53 | 0.45 | 0.42 | 0.07 | 0.29 |
| | YES | 56 | 0.57 ± 0.25 | 0.62 ± 0.26 | 0.49 ± 0.27 | 0.58 ± 0.22 | 0.62 ± 0.26 | 0.61 ± 0.27 | 0.51 ± 0.3 | 0.48 ± 0.23 | 0.59 ± 0.22 |
| | NO | 555 | 0.53 ± 0.23 | 0.59 ± 0.26 | 0.44 ± 0.26 | 0.54 ± 0.21 | 0.59 ± 0.26 | 0.59 ± 0.26 | 0.47 ± 0.28 | 0.42 ± 0.21 | 0.56 ± 0.21 |
| | **Total** | 611 | 0.53 ± 0.23 | 0.59 ± 0.26 | 0.44 ± 0.26 | 0.55 ± 0.21 | 0.59 ± 0.26 | 0.59 ± 0.26 | 0.47 ± 0.28 | 0.42 ± 0.22 | 0.57 ± 0.21 |
| Cardiovascular | | | 0.78 | 0.36 | 0.65 | 0.85 | 0.34 | 0.68 | 0.24 | 0.99 | 0.72 |
| | YES | 7 | 0.49 ± 0.21 | 0.69 ± 0.26 | 0.39 ± 0.33 | 0.56 ± 0.21 | 0.7 ± 0.25 | 0.54 ± 0.23 | 0.62 ± 0.3 | 0.41 ± 0.22 | 0.6 ± 0.19 |
| | NO | 589 | 0.53 ± 0.23 | 0.59 ± 0.26 | 0.44 ± 0.26 | 0.55 ± 0.21 | 0.59 ± 0.26 | 0.59 ± 0.26 | 0.47 ± 0.28 | 0.42 ± 0.22 | 0.56 ± 0.21 |
| | **Total** | 596 | 0.53 ± 0.23 | 0.59 ± 0.26 | 0.44 ± 0.26 | 0.55 ± 0.21 | 0.59 ± 0.26 | 0.59 ± 0.26 | 0.47 ± 0.28 | 0.42 ± 0.22 | 0.56 ± 0.21 |
| Cholesterol | | | 0.16 | 0.51 | 0.74 | 0.66 | 0.51 | 0.17 | 0.69 | 0.61 | 0.85 |
| | YES | 32 | 0.58 ± 0.2 | 0.57 ± 0.22 | 0.46 ± 0.26 | 0.57 ± 0.19 | 0.57 ± 0.22 | 0.65 ± 0.23 | 0.49 ± 0.29 | 0.41 ± 0.22 | 0.58 ± 0.19 |
| | NO | 563 | 0.53 ± 0.23 | 0.59 ± 0.26 | 0.44 ± 0.26 | 0.55 ± 0.21 | 0.59 ± 0.26 | 0.59 ± 0.26 | 0.47 ± 0.28 | 0.42 ± 0.22 | 0.56 ± 0.21 |
| | **Total** | 595 | 0.53 ± 0.23 | 0.59 ± 0.26 | 0.44 ± 0.26 | 0.55 ± 0.21 | 0.59 ± 0.26 | 0.59 ± 0.26 | 0.47 ± 0.28 | 0.42 ± 0.22 | 0.56 ± 0.21 |
| Psychic disorders | | | 0.03 | 0.17 | 0.04 | 0.05 | 0.19 | 0.07 | 0.72 | 0.005 | 0.07 |
| | YES | 175 | 0.56 ± 0.23 | 0.62 ± 0.25 | 0.48 ± 0.25 | 0.57 ± 0.2 | 0.62 ± 0.25 | 0.62 ± 0.26 | 0.47 ± 0.29 | 0.46 ± 0.21 | 0.59 ± 0.2 |
| | NO | 436 | 0.52 ± 0.23 | 0.59 ± 0.26 | 0.43 ± 0.26 | 0.54 ± 0.21 | 0.58 ± 0.26 | 0.58 ± 0.25 | 0.48 ± 0.28 | 0.41 ± 0.22 | 0.56 ± 0.21 |
| | **Total** | 611 | 0.53 ± 0.23 | 0.59 ± 0.26 | 0.44 ± 0.26 | 0.55 ± 0.21 | 0.59 ± 0.26 | 0.59 ± 0.26 | 0.47 ± 0.28 | 0.42 ± 0.22 | 0.57 ± 0.21 |

P values from Mann-Whitney test.

2.4 Latent Class Analysis

The LCA selected 7 more information items, as reported in Figure 2. Subjects were then classified into four classes, according to their responses to the selected items. For each class, Figure 2 shows the percentages of responses to each item (from 0 to 4, Likert scale). Class 1 was made up of women who perceived the presence of air, water, and food pollution and who responded with the highest degree of the scale in relation to the perception of bad smells, dangerous industries, and in relation to the presence of chronic respiratory diseases. Class 2 and Class 3 were mainly composed of women who responded to the different items with a degree between "enough" and "very", and between "a little" and "enough", respectively. Class 4 contained women who perceived a low degree of exposure to the different risks, with the largest percentages of responses to the items between "not at all" and "little". In summary, women in Class 1 were those that felt more exposed to the selected risks, followed by the women in Class 2, Class 3 and Class 4.

The RP indices were significantly different among the four classes, increasing as exposure risk perception increased. The same results were obtained for the EFA factors, except for Factor 3 (traffic exposure), which was not significantly different in the four classes (Table S4). The NPCS variable (the three sites) was significantly associated with the classification (P<0.001). While in the Priolo and Crotone sites mothers showed a similar distribution profile, with the highest percentages in the intermediate classes (classes 2 and 3) and the lowest percentages in the extreme classes (classes 1 and 4), inhabitants of the Milazzo site presented a fairly homogeneous distribution in the first three classes, with a very small percentage of women (1.2%) in the class with the smallest degree of exposure risk perception (Class 4). Association between the class variable and Area (at-risk versus LRA) was also significant (P<0.001), with similar percentages of women in the first three classes and a low percentage in Class 4 for the at-risk area group. On the contrary, women in the LRAs were distributed more in the intermediate classes, with the lowest percentage in the class at highest degree of risk exposure perception. Regarding educational level, the percentage of women with the highest degree of qualification and belonging to Class 4 was lower than the percentages in the other three classes (P=0.016) (Table 5).

Figure 3 shows the geographical distribution of mothers in each NPCS. The Figure shows that, in the Priolo NPCS, most of the mothers living in the at-risk area belonged to the medium/high risk perception classes, while most of the mothers living in the LRA were in the low/medium class. In Crotone, the four classes were homogeneously distributed between the NPCS and LRA areas, while in Milazzo only one mother perceived a low risk, with the rest equally distributed in the other classes, independently from their residence area.

**Supplementary materials Table S4.** Average mean and standard deviation of the Risk Perception and Exploratory Factor Analysis indices, according to the classes from the Latent Class Analysis.

| INDEX | | N | mean (±sd) | p.value |
|---|---|---|---|---|
| EHPI | Class 1 | 105 | 0.83 ± 0.1 | <0.001 |
| | Class 2 | 209 | 0.66 ± 0.09 | |
| | Class 3 | 224 | 0.4 ± 0.09 | |
| | Class 4 | 73 | 0.15 ± 0.09 | |
| | **Total** | **611** | **0.53 ± 0.23** | |
| HRPI | Class 1 | 105 | 0.76 ± 0.24 | <0.001 |
| | Class 2 | 209 | 0.67 ± 0.2 | |
| | Class 3 | 224 | 0.53 ± 0.23 | |
| | Class 4 | 73 | 0.34 ± 0.26 | |

| | | | | |
|---|---|---|---|---|
| | **Total** | **611** | **0.59 ± 0.26** | |
| **HPI** | Class 1 | 105 | 0.64 ± 0.25 | <0.001 |
| | Class 2 | 209 | 0.52 ± 0.23 | |
| | Class 3 | 224 | 0.36 ± 0.21 | |
| | Class 4 | 73 | 0.19 ± 0.17 | |
| | **Total** | **611** | **0.44 ± 0.26** | |
| **RPI** | Class 1 | 105 | 0.78 ± 0.13 | <0.001 |
| | Class 2 | 209 | 0.65 ± 0.11 | |
| | Class 3 | 224 | 0.45 ± 0.12 | |
| | Class 4 | 73 | 0.23 ± 0.12 | |
| | **Total** | **611** | **0.55 ± 0.21** | |
| **FACTOR 1** | Class 1 | 105 | 0.76 ± 0.24 | <0.001 |
| | Class 2 | 209 | 0.67 ± 0.2 | |
| | Class 3 | 224 | 0.52 ± 0.23 | |
| | Class 4 | 73 | 0.34 ± 0.25 | |
| | **Total** | **611** | **0.59 ± 0.26** | |
| **FACTOR 2** | Class 1 | 105 | 0.89 ± 0.13 | <0.001 |
| | Class 2 | 209 | 0.74 ± 0.11 | |
| | Class 3 | 224 | 0.45 ± 0.11 | |
| | Class 4 | 73 | 0.16 ± 0.1 | |
| | **Total** | **611** | **0.59 ± 0.26** | |
| **FACTOR 3** | Class 1 | 100 | 0.49 ± 0.32 | 0.01 |
| | Class 2 | 203 | 0.49 ± 0.28 | |
| | Class 3 | 213 | 0.49 ± 0.25 | |
| | Class 4 | 73 | 0.37 ± 0.32 | |
| | **Total** | **589** | **0.47 ± 0.28** | |
| **FACTOR 4** | Class 1 | 105 | 0.67 ± 0.17 | <0.001 |
| | Class 2 | 209 | 0.47 ± 0.18 | |
| | Class 3 | 224 | 0.32 ± 0.15 | |
| | Class 4 | 73 | 0.23 ± 0.16 | |
| | **Total** | **611** | **0.42 ± 0.22** | |
| **EFA_TOT** | Class 1 | 105 | 0.80 ± 0.13 | <0.001 |
| | Class 2 | 209 | 0.67 ± 0.11 | |
| | Class 3 | 224 | 0.47 ± 0.12 | |
| | Class 4 | 73 | 0.25 ± 0.12 | |
| | **Total** | **611** | **0.57 ± 0.21** | |

P value for differences among classes by one-way ANOVA. EHPI: Exposure Hazard Perception Index; HRPI: the Health Risk Perception Index; HPI: Hazard Perception Index; RPI Risk Perception Index. FACTOR 1-4: Exploratory Factor Analysis factors.
